# Data linkage research to explore the growing share of domestic abuse cases in family courts

**DOI:** 10.23889/ijpds.v8i2.2205

**Published:** 2023-09-14

**Authors:** Ludivine Garside

**Affiliations:** 1 University of Bristol, Bristol, United Kingdom

## Objectives

The caseload of family courts in England and Wales includes a growing share of domestic abuse cases. To increase knowledge about such cases, the research uses new administrative datasets and linkages to describe trends between 2011-2021 and to supplement the information already available from family court national statistics.

## Method

The complete set of family court cases is held in HM Courts & Tribunals Services (HMCTS) systems. Some family court data are collected by the Children and Families Court Advisory Support Service (Cafcass), insofar as courts refer the case to Cafcass. By using their new linkage, we can conduct a descriptive analysis of domestic abuse cases in England and Wales over the ten years since 2011. This project is one of the first to gain approval from HMCTS to use their family court data. Access to de-identified versions of all datasets is through the SAIL Databank trusted research environment.

## Results

The data linkage builds a more complete picture of the individuals involved, exploring their age group, gender and ethnicity. The new datasets allow to describe how court users approach domestic abuse matters and to draw distinctions between different types of cases: emergency situations; individuals being unrepresented in court (litigants in person); repeat applications; requests for different types of legal measures against the domestic abuse; and presence of children in the household. Furthermore, the data show how courts respond through case management, timeliness of decisions and legal outcomes made. We explore how different types of individuals and cases are associated with legal outcomes, and how the added understanding at population-level can focus and support discussions about the use of family courts to address domestic abuse.

## Conclusion

Data linkage research is providing an evidence base at population level about court users in England and Wales who experience domestic abuse. This can help inform practice to support those families and policies to ease overburdened courts who see the same families and domestic abuse issues return to court.

